# Disseminated Tuberculosis of the Lungs, Brain, Pleurae, Mediastinal Lymphadenopathy, and Elbow Joint in an Immunocompetent Indian Female: A First-of-Its-Type Case

**DOI:** 10.7759/cureus.58974

**Published:** 2024-04-25

**Authors:** Sankalp Yadav, Surinder Pal, Gautam Rawal, Madhan Jeyaraman, Naveen Jeyaraman

**Affiliations:** 1 Medicine, Shri Madan Lal Khurana Chest Clinic, New Delhi, IND; 2 Medicine, Choudhary Desraj Chest Clinic, New Delhi, IND; 3 Respiratory Medical Critical Care, Max Super Speciality Hospital, New Delhi, IND; 4 Clinical Research, Virginia Tech India, Dr. MGR Educational and Research Institute, Chennai, IND; 5 Orthopedics, ACS Medical College and Hospital, Dr. MGR Educational and Research Institute, Chennai, IND

**Keywords:** cartridge-based nucleic acid amplification test (cbnaat), mediastinal lymphadenopathy, pleural effusion, disseminated tuberculosis, mycobacterium tuberculosis (mtb), intracranial tuberculoma, tuberculoma

## Abstract

Tuberculosis is usually seen in the lungs. However, the involvement of various extrapulmonary sites is due to the spread of the bacteria via blood, lymphatic, or direct inoculation. The present case is a rare presentation of tuberculosis in an Indian female who came with complaints of swelling in her right elbow joint, headache, and cough with expectoration. A diagnostic evaluation resulted in the isolation of *Mycobacterium tuberculosis* from the sputum samples and elbow joints, which was further supported by an exudative picture on the cerebrospinal fluid examination. The findings were supported by advanced radiometric techniques. She was commenced on an antituberculous treatment per her weight. Disseminated tuberculosis is a challenging diagnosis as there is often a delay in clinical presentation, a lack of awareness about the possibility of multiple sites with tuberculous infection in clinicians, and a time lag in the availability of the culture results.

## Introduction

Tuberculosis is a clinical outcome of infection by *Mycobacterium tuberculosis* [1]. This bacteria has a proclivity to affect the lungs, but sites other than the lungs contribute about 10%-15% of total tuberculosis cases [2]. This disease is a significant cause of mortality and morbidity in endemic countries. With an incidence of 188 and a prevalence of 312 per 0.1 million population, India has a significant burden of the disease [2,3].

The progressive, potentially fatal illness known as "miliary" or "disseminated" tuberculosis arises from the lymphohematogenous spread of *Mycobacterium tuberculosis*, either as a result of primary dissemination or as a result of the reactivation of dormant tuberculosis foci, or it could be iatrogenic [2,4]. It is a rare clinical condition, with about 1%-2% of all tuberculosis cases being of the disseminated type [4].

We report a rare case of disseminated tuberculosis of the lungs, brain, pleurae, mediastinal lymphadenopathy, and elbow joint in an immunocompetent Indian female. A diagnosis was challenging as suspecting concurrent infection of noncontiguous sites in the same patient is exceedingly rare. A detailed search of the medical literature revealed that a similar case of a common disease with the atypical involvement of multiple sites such as the pleurae, mediastinal lymph nodes, elbow joint, lungs, and brain has never been documented before.

## Case presentation

A 29-year-old nondiabetic Indian female came as a referral case from a nearby government center. She came with chief complaints of nausea and vomiting (two episodes in 24 hours), pain and swelling in her right elbow joint for three months, fever without chills off and on for two months, cough with expectoration, and generalized headache for two months. She also complained of a loss of appetite and weight (5 kg in three months).

She was apparently well three months ago when she had pain and swelling over her right elbow joint. The swelling was insidious in onset and was not associated with any discharge. She had a fever that rose in the evening and was relieved after taking over-the-counter paracetamol. Further, she had a cough that was associated with a yellow-colored, no-foul-smelling, non-blood-tinged expectoration for two months. Furthermore, she had a generalized headache that was persistent for two months. She also had two episodes of non-projectile vomiting in 24 hours.

There was no history of trauma, chest pain, night sweats, seizures, loss of consciousness, or any contact with tuberculosis in the family. Further, she was a housewife from a high-income group background with no history of substance abuse or staying in crowded places.

A general examination was suggestive of a hemodynamically stable young female with an ectomorphic build with a Glasgow Coma Scale of 15/15. There was no pallor, cyanosis, clubbing, pedal edema, or icterus. However, her systemic examination was suggestive of bilateral bronchophony or pectoriloquy on auscultation of the lungs. On a central nervous system examination, the pupils were 3 mm, equally reactive bilaterally. Power was 5/5 in all muscle groups except in the right upper limb, where it was 3/5. The sensory and cerebellar examinations were unremarkable. Neck rigidity was absent with negative Kernig's and Brudzinski's signs. Fundoscopy was unremarkable. All other examinations were unremarkable.

A local examination of the right elbow joint was notable for a tender-to-touch, swollen joint with a reduced range of movement. However, there was no erythema or discharging sinus. A Bacillus Calmette-Guérin (BCG) scar was visible. The left elbow joint was normal.

With the majority of constitutional symptoms of tuberculosis in an endemic country, a provisional diagnosis of tuberculosis was made, and a detailed laboratory workup was initiated. The results of her laboratory investigations were remarkable for a raised erythrocyte sedimentation rate (71 mm/hour), C-reactive proteins (8.0 mg/dL), and a strongly positive Mantoux test (20 × 20 mm induration). The rest of the tests, including HIV, hepatitis panel, and rheumatoid factor, were negative. A sputum test for acid-fast bacillus was negative. But a cartridge-based nucleic acid amplification test detected a very low amount of *Mycobacterium tuberculosis* without any resistance to rifampicin.

**Figure 1:**
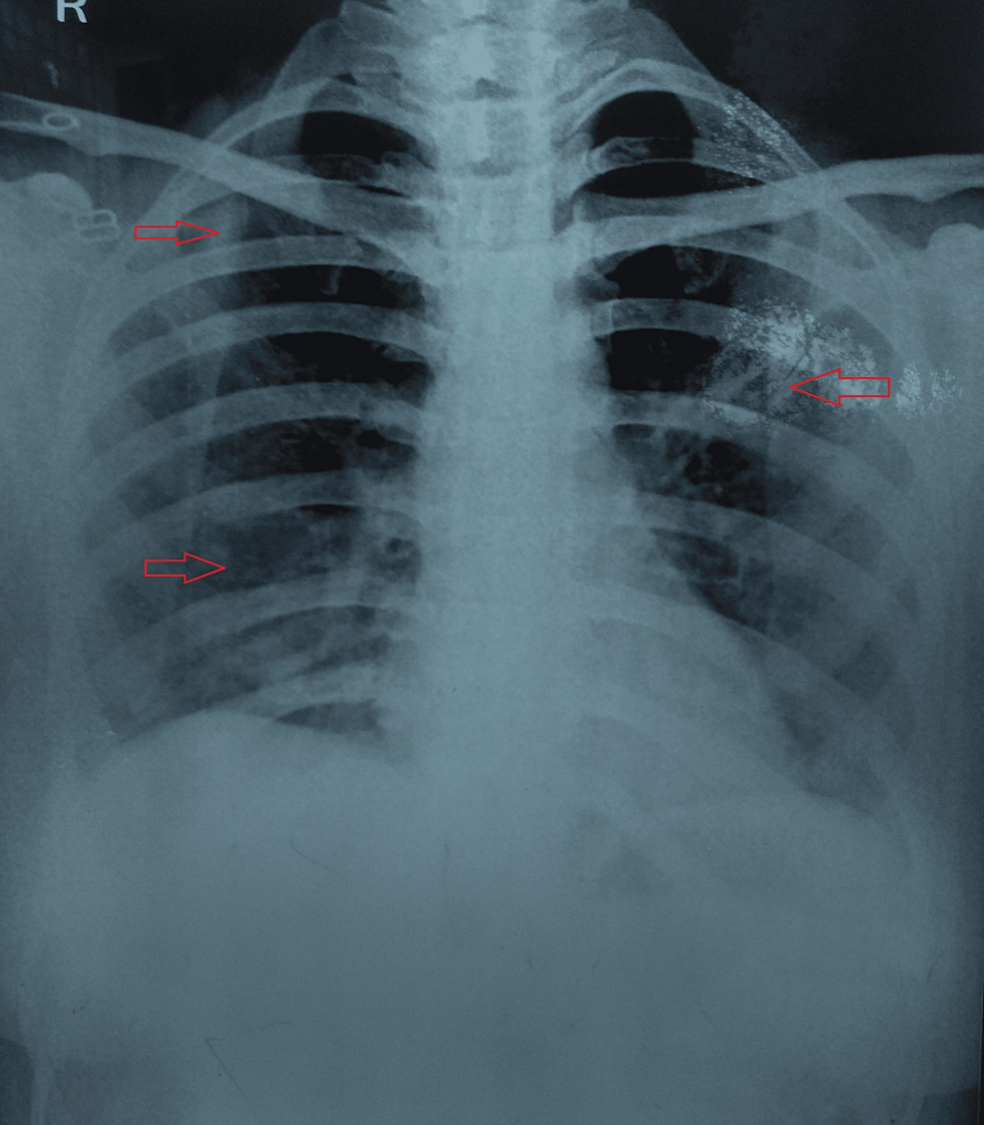
Plain chest radiograph suggestive of bilateral patchy consolidations

Her chest radiograph was suggestive of bilateral patchy consolidations (Figure 1).

**Figure 2:**
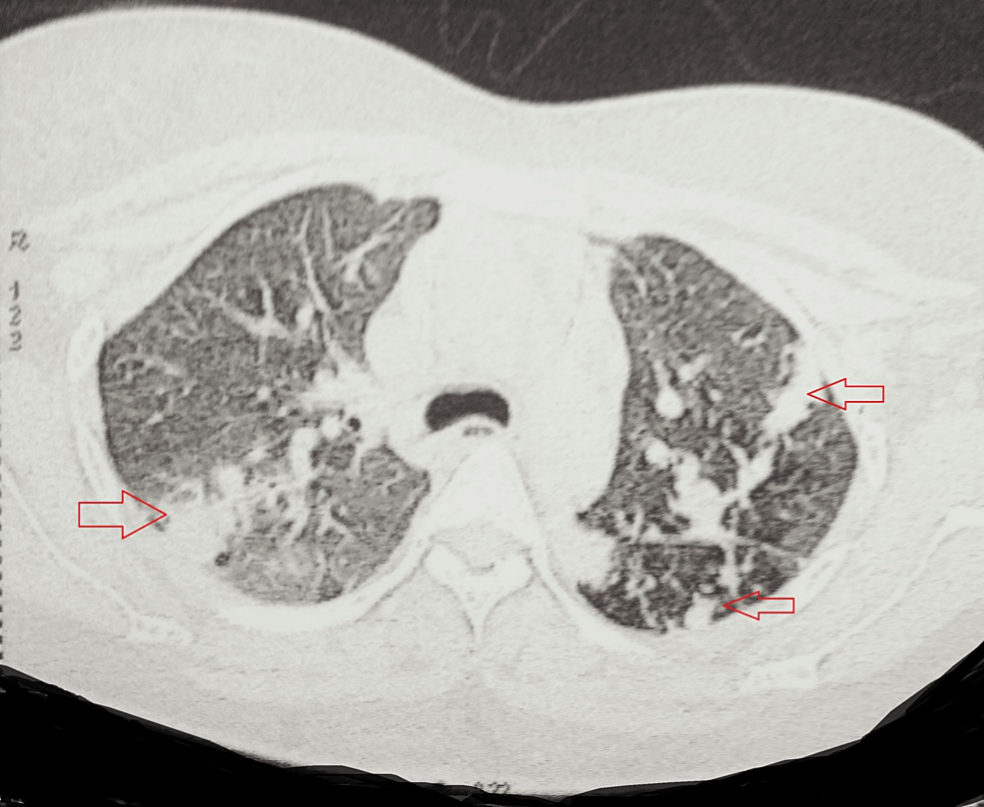
HRCT of the chest showing multiple bilateral patchy consolidations HRCT: high-resolution computed tomography

**Figure 3:**
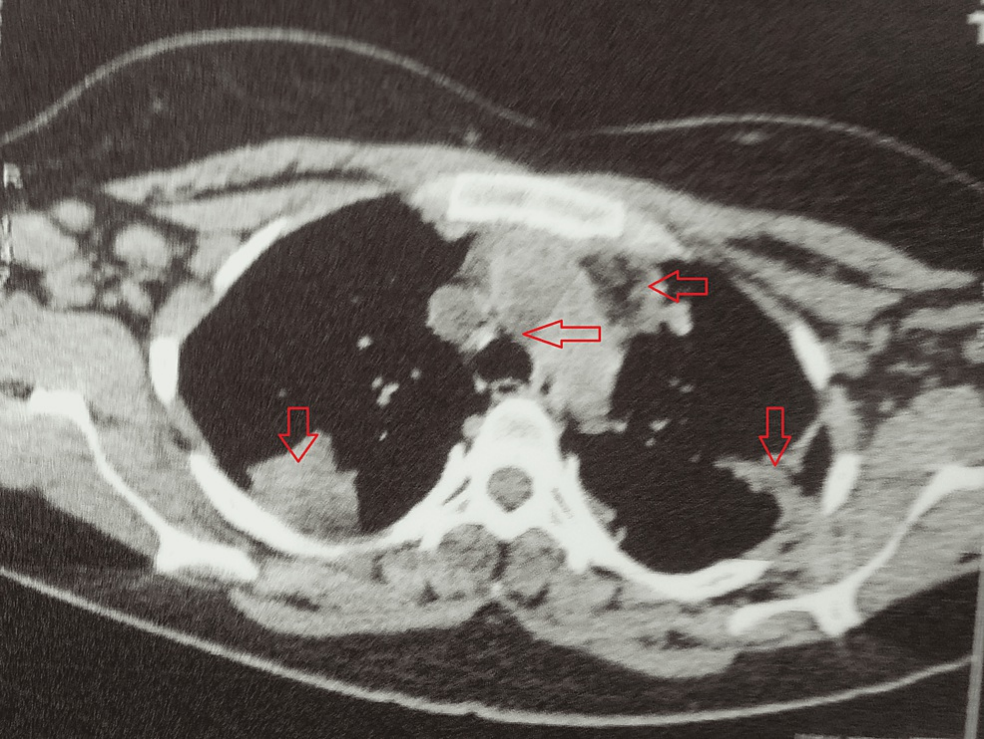
HRCT of the chest showing mediastinal lymphadenopathy and lung involvement HRCT: high-resolution computed tomography

A high-resolution computed tomography of the chest revealed multiple bilateral patchy consolidations in the lung parenchyma, exhibiting breakdown areas and an air bronchogram within. Also, confluent fibronodular lesions were widely scattered in both lungs, with interspersed ground glassing and thickened intervening inter- and intralobular septa. Mild bilateral pleural effusion was seen with diffusely thickened adjoining pleurae. Besides, there were evidently enlarged mediastinal lymph nodes, i.e., prevascular, pre-/subcarinal, and bilateral hilar lymph nodes (Figures 2, 3).

**Figure 4:**
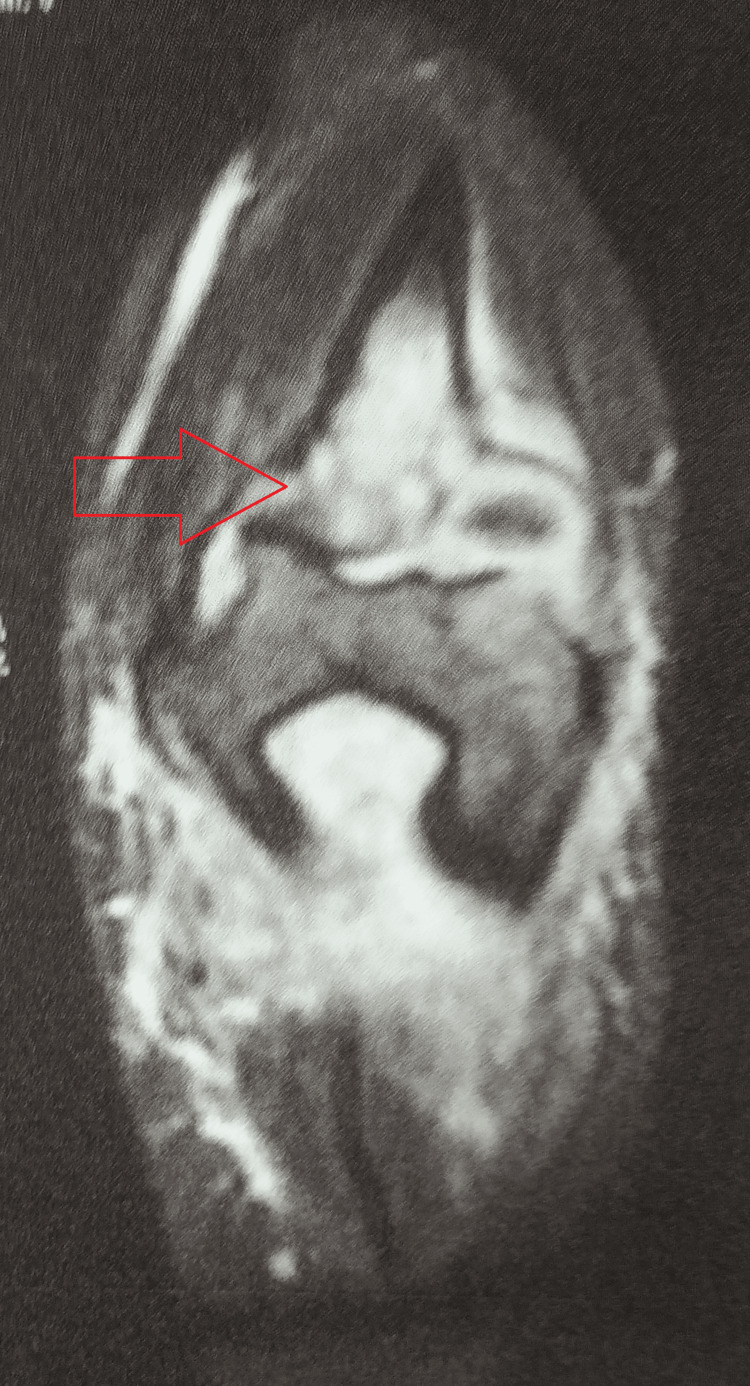
MRI of the right elbow joint suggestive of subarticular erosive changes in the bones forming the elbow joint MRI: magnetic resonance imaging

Magnetic resonance imaging of the right elbow joint was suggestive of subarticular erosive changes in the bones forming the elbow joint, including the distal humerus, contiguous radius, and ulna, with diffuse underlying marrow edema appearing hyperintense on T2 and short tau inversion recovery (STIR) images. Moderate joint effusion was seen, with diffuse thickening of the underlying synovia. Periarticular soft tissues were diffusely thickened and edematous, with multiple loculi of heterogenous fluid collections noted along intervening inter-/intramuscular and fascial planes and enlarged locoregional lymph nodes (Figure 4).

**Figure 5:**
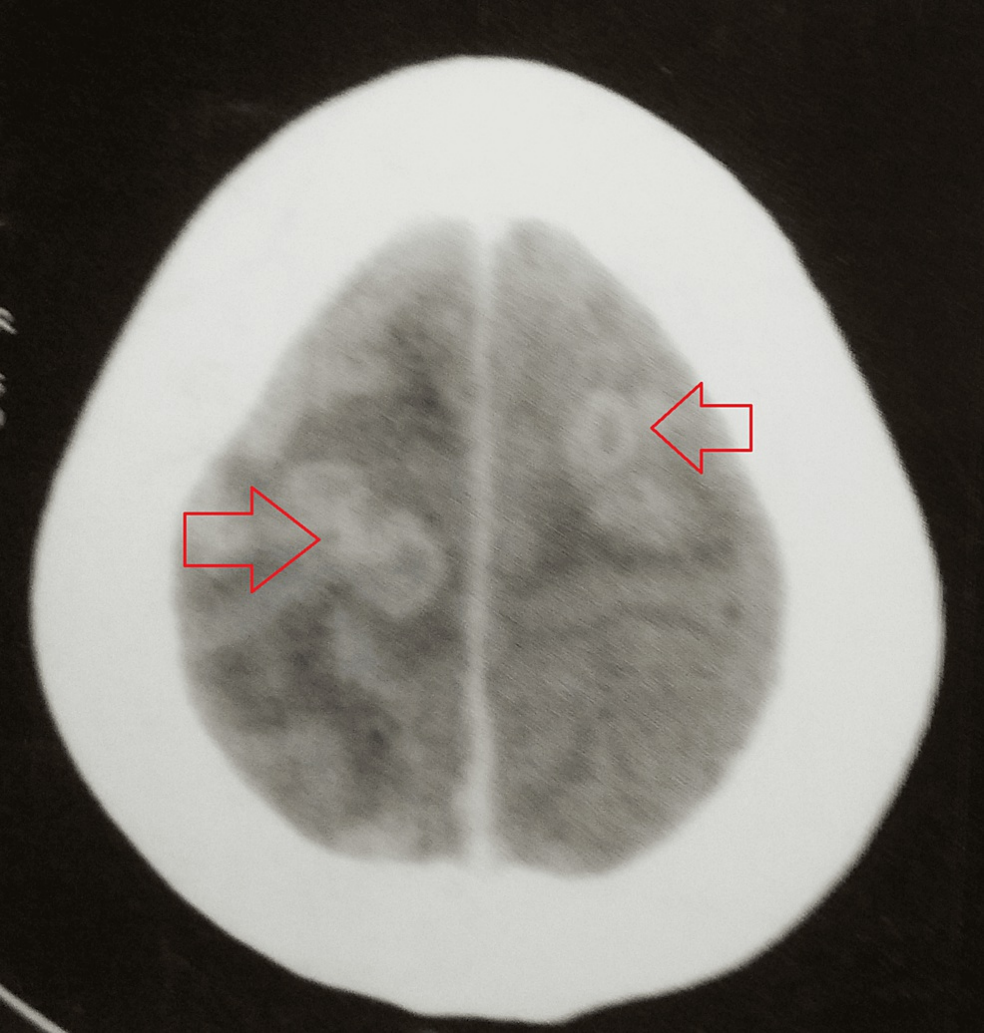
CECT of the brain showing well-defined, multiple discrete and conglomerated ring-enhancing lesions scattered in the bilateral cerebellar hemisphere CECT: contrast-enhanced computed tomography

**Figure 6:**
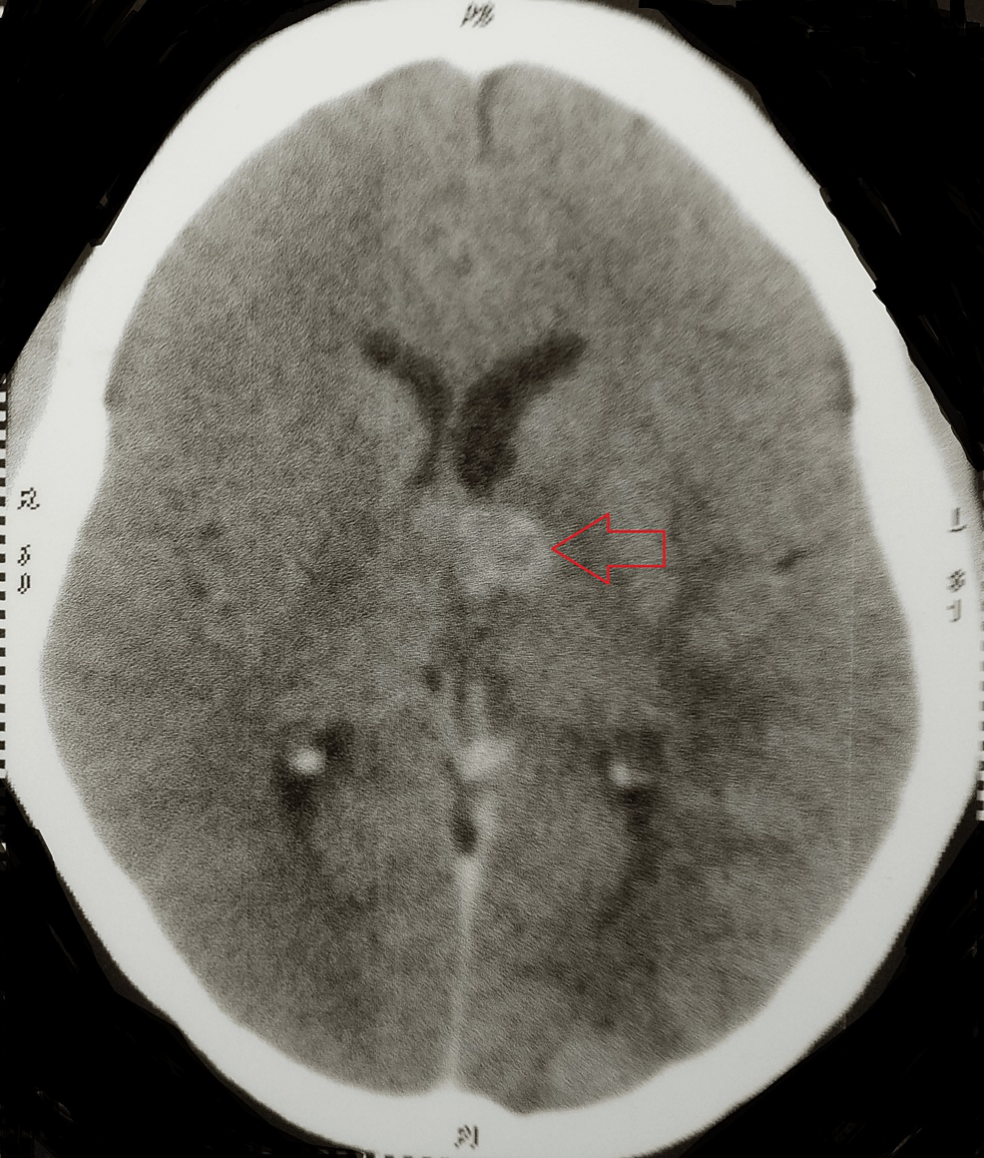
CECT of the brain showing a well-defined, discrete ring-enhancing lesion in the left thalamus CECT: contrast-enhanced computed tomography

A contrast-enhanced computed tomography of the brain showed well-defined, multiple discrete and conglomerated ring-enhancing lesions scattered in the bilateral cerebellar hemisphere and left thalamus, with mild to moderate surrounding perilesional edema. There was associated leptomeningeal enhancement in the right high parietal region, likely inflammatory granulomas or tuberculomas (Figures 5, 6).

An ultrasound-guided thoracentesis of the pleurae was a dry tap. An aspiration of the joint fluid was done in sterile conditions in an orthopedic minor operation theater, and it was sent for microscopy, a cartridge-based nucleic acid amplification test, and culture. The results were remarkable for the very low detection of *Mycobacterium tuberculosis* with no rifampicin resistance on a cartridge-based nucleic acid amplification test. All the other tests, including a line-probe assay of the joint fluid and culture, were negative.

A lumbar puncture was done, and the findings were suggestive of an exudate (lymphocytic predominance with elevated proteins and low glucose levels). A final diagnosis of disseminated tuberculosis of the lungs, brain, pleurae, mediastinal lymph nodes, and elbow joint in an immunocompetent Indian female was made on the basis of the detection of *Mycobacterium tuberculosis* in the sputum and elbow joint fluid along with the radiological evidence and exudative picture on lumbar puncture, and she was commenced on an antituberculous treatment per the national guidelines with an initiation phase of fixed-dose combinations of four drugs, i.e., isoniazid, ethambutol, rifampicin, and pyrazinamide, in the initiation phase of two months and 10 months of the continuation phase with three drugs, i.e., isoniazid, rifampicin, and ethambutol [5].

She gained weight (about 3 kg) and did well for the three months without experiencing any negative drug effects. Later, she requested to be transferred to her hometown, and her request was granted. She received advice on adhering to her treatment plan, eating a diet high in protein, and scheduling routine checkups at the closest clinic for infectious diseases, neurology, and orthopedics outpatient departments.

## Discussion

Disseminated tuberculosis is an infrequently reported condition [2,4]. The same is evident by the prevalence of less than 2% of total cases of tuberculosis in immunocompetent adults, which is about 20% of all extrapulmonary tuberculosis cases [6]. A higher incidence of disseminated and pulmonary tuberculosis is reported in males [7].

Although disseminated tuberculosis is relatively uncommon, elderly patients; persons with childhood diseases, HIV, alcoholism, diabetes, chronic liver or kidney failure, and organ transplants; patients on pharmacological immunosuppressive drugs; pregnant patients; and those having symptoms lasting longer than 12 weeks are among the predisposing factors to disseminated tuberculosis [4,8].

*Mycobacterium tuberculosis* can disseminate to any of the organs in the body. However, about 22% involve the central nervous system, manifesting as meningitis, cerebral tuberculoma, tuberculoma abscess, and thoracic transverse myelopathy [4]. The present case had cerebral tuberculomas.

Further, in all age groups, the disease has substantial morbidity and mortality [2]. Anorexia, nocturnal sweats, exhaustion, hemoptysis, headache, dyspnea, fever, mental abnormalities, ascites, pleural effusion, and lymphadenopathy are the most prevalent clinical characteristics in adults [8].

The diagnosis can be challenging due to ambiguity in the clinical presentations [2]. This could be attributed to the paucity of tools available for quick laboratory diagnosis; in addition, samples are often paucibacillary, the acid-fast bacilli smear has low sensitivity, cultures take a long time, and there are nonspecific findings on chest radiographs [4]. Additionally, the clinical presentation of this condition is hard to determine and vague [8].

The diagnosis is mainly based on the isolation of *Mycobacterium tuberculosis* on culture [2]. However, it takes a significant amount of time and could be negative, as seen in the present cases [9]. A timely diagnosis is pertinent for a favorable outcome; hence, a detailed clinical assessment with an adjunct of advanced radiometric investigations is imperative [2]. The same was noted in the present case.

Management is essentially conservative [2,5]. The guidelines for the National Tuberculosis Elimination Programme are the base of management for such cases in India. For drug-sensitive extrapulmonary tuberculosis such as the bones and central nervous system, a 12-month treatment with a clinical assessment at the end of treatment with the possibility to extend the treatment is mentioned [5]. Surgical interventions are used in cases where there is a loss of joint movement or to remove the sequestrum [4]. However, for tuberculomas, surgery is rarely indicated. Ruling out drug resistance is essential, as cases of primary disseminated tuberculosis are documented in the literature [5,10]. A case similar to this was presented by Yadav (2023); the present case differs in terms of gender, age, and the absence of the involvement of the elbow joints and brain; and there was no pericardium involvement in the present case [2].

A rare case of disseminated tuberculosis of the lungs, brain, pleurae, mediastinal lymph nodes, and elbow joint in an immunocompetent Indian female is reported here. The limitation is that it was only one case, and hence, the management protocol was per the general guidelines.

## Conclusions

A first-of-its-type case of disseminated tuberculosis of the lungs, pleurae, mediastinal lymph nodes, brain, and elbow joint in an immunocompetent Indian female is reported here. The case highlights the importance of timely diagnosis, as the ambiguity around the clinical presentations could result in fatal outcomes. Moreover, the paucity of data related to disseminated tuberculosis emphasizes the dissemination of information about such rare concomitant infections of a very common infection.

## References

[1] Zaman K (2010). Tuberculosis: a global health problem. J Health Popul Nutr.

[2] Yadav S (2023). Disseminated tuberculosis of the lungs, pleura, mediastinal lymph nodes, and pericardium: a rare case report. Cureus.

[3] (2024). Global tuberculosis report 2023. https://www.who.int/teams/global-tuberculosis-programme/tb-reports/global-tuberculosis-report-2023.

[4] Esposito SB, Levi J, Matuzsan ZM, Amaducci AM, Richardson DM (2020). A case report of widely disseminated tuberculosis in immunocompetent adult male. Clin Pract Cases Emerg Med.

[5] (2024). Training module on extrapulmonary tuberculosis 2023. https://tbcindia.gov.in/WriteReadData/l892s/7702334778Training_Module_on_Extrapulmonary_TB_-_Book_24032023.pdf.

[6] Sharma SK, Mohan A, Sharma A (2016). Miliary tuberculosis: a new look at an old foe. J Clin Tuberc Other Mycobact Dis.

[7] Fidalgo M, Cabral J, Soares I, Oliveira M (2023). From testicle to brain: a case of disseminated tuberculosis. Cureus.

[8] Khan FY (2019). Review of literature on disseminated tuberculosis with emphasis on the focused diagnostic workup. J Family Community Med.

[9] Campelo TA, Cardoso de Sousa PR, Nogueira LL, Frota CC, Zuquim Antas PR (2021). Revisiting the methods for detecting Mycobacterium tuberculosis: what has the new millennium brought thus far?. Access Microbiol.

[10] Yadav S (2023). Primary disseminated pre-extensively drug-resistant tuberculosis of the lungs, pleura, chest wall, and abdomen: the world’s first case. Cureus.

